# Scores and scales used in emergency medicine. Practicability in toxicology

**Published:** 2014

**Authors:** B Oprita, B Aignatoaie, DA Gabor-Postole

**Affiliations:** * “Floreasca” Clinical Emergency Hospital of Bucharest, Bucharest, Romania

**Keywords:** medical score, PSS, AIS, ISS, CRAMS

## Abstract

Medical scores, criteria and classification systems support clinical decision-making and management. They enable the clinician to predict the outcome, stratify risk, assess conditions and diagnose diseases accurately.

In the emergency medicine, it is very important to ascertain safety criteria to discharge patients, time to remain in the E.R., and also ascertain the time intervals for discharge/admission.

The use of the scores in the emergency medicine, toxicology and other areas of intensive medicine have become increasingly efficient.

Creating a prognostic score for the acute intoxications to be used by the personnel from the Emergency Departments may have positive effects in the management of the poisoned patients (e.g. the admission in a certain treatment space: cases expected to have a trend towards worsening will be directed to the resuscitation space and after a short period of time admitted in the appropriate facility; this way, the bed occupancy time in the Emergency Department will be shortened).

## Introduction

Medical scores, criteria and classification systems support clinical decision-making and management. They enable the clinician to predict the outcome, stratify risk, assess conditions and diagnose diseases accurately [**1**].

In the emergency medicine, it is very important to ascertain safety criteria to discharge patients, time to remain in the E.R., and also ascertain the time intervals for discharge/ admission. Several scoring systems for the assessment of the severity of illness have been presented during the last decades.

They have mainly been directed to the critically ill, and their common purpose was to measure the deviations in different physiological variables, in order to provide an objective measurement of the severity of the illness recognized by physicians worldwide.

## Method

From the many scores used in Emergency Medicine, Intensive Care and Toxicology, the ones with a wide extension and with proved efficiency through time, were selected. The parameters calculated upon them were analyzed and the communicated information regarding their reliability and limitations was studied. 

The ideal scoring system would have the following characteristics:

1. On the basis of easily/ routinely recordable variables

2. Well calibrated

3. A high level of discrimination

4. Applicable to all patient populations

5. Can be used in different countries

6. The ability to predict functional status or quality of life after ICU discharge [**4**]

No scoring system currently incorporates all these features. There is no agreed classification of the scoring systems that are used in critically ill patients. Scores can be applied either to a single set of data or repeated over time [**4**]. 

The available methods include:

***1. Anatomical scoring.***

a. These depend on the anatomical area involved. Anatomical scoring systems are mainly used for trauma patients [e.g. abbreviated injury score (AIS) and injury severity score (ISS)]. 

**Abbreviated Injury Scale (AIS)** measures only the anatomic injury. AIS was originally intended for the documentation of the motor vehicle accidents. It comprises a lexicon of six-digit coded injuries (>2,000 listed) based on: region, type of anatomic structure, specific structure, level of injury. Injuries are assigned a severity value of 1 (minor) to 6 (fatal). 

b. **Injury Severity Score (ISS; 1974)** represents the summation of AIS scores from the squared values of the three most severely injured body areas. The scores ranges are from 0 to 75. Benefits: includes more injuries in its score derivation.

2. ***Therapeutic weighted scores***. These are based on the assumption that very ill patients require a greater number of interventions and procedures that are more complex, than patients who are less ill. Examples include the therapeutic intervention scoring system (TISS). 

3. ***Organ-specific scoring***. This is similar to the therapeutic scoring; the underlying premise is the sicker a patient, the more organ systems will be involved, ranging from organ dysfunction to failure [e.g. sepsis-related organ failure assessment (SOFA)]. 

4. ***Physiological assessment***. It is based on the degree of derangement of routinely measured physiological variables [e.g. acute physiology and chronic health evaluation (APACHE) and simplified acute physiology score (SAPS)].

APACHE III (1992) is intended to rectify trauma limitation but the system is proprietary.

It is more complex than the other commonly available ICU admission scoring systems [**4**].

The APACHE II uses a point score based upon values of 12 routine physiological measurements as well as age and previous health status. The variables included in the APACHE II system are body temperature, mean arterial pressure calculated according to the systolic and diastolic blood pressure, heart rate, oxygenation of arterial blood (PaO2), arterial pH, serum sodium, serum potassium, serum creatinine, hematocrit, white blood count and GCS. 

The maximal APACHE II score is 71. 

Benefits:

• Drotrecogin alpha may be considered for the treatment of sepsis based on the presence of both:

o Sepsis-induced multiple organ failure, septic shock, or sepsis-induced acute respiratory distress syndrome [ARDS]) and,

o An APACHE II score >25 [**4**]

**Simplified Acute Physiology Score (SAPS II; 1993)**

• Similar to APACHE II 

o 12 routine physiological measurements during the first 24 hours, age, and prior health status

o Score ranges from 0-24 [**4**].

5. ***Simple scales***. They are based on clinical judgment (e.g. survive or die).

Glasgow coma scale (GCS) first appeared in 1974 in reports by Graham Teasdale and Bryan J. Jennet, both professors of neurosurgery at the University of Glasgow. GCS is now used as an indicator for the status of the central nervous system regardless of its primary etiology. Poisoning with drugs influences the biochemical substances of the brain and causes brain damage. This may change the level of consciousness as well. The GCS has been performed for the outcome and recovery evaluation of patients admitted to an intensive care unit (ICU) following drug overdose, for the mental status evaluation of the poisoned patients, the need for intubation in patients with antidepressant poisoning, and for predicting acute and delayed poisoning outcomes. All previous studies showed that for the assessment of complication and mortality, the GCS score provides the best indicator in poisoned patients; however, no study was done to show the way the prediction is the subset of GCS (eye, motor, and verbal). Mixed drugs poisoning (MDP) is one of the most common reasons for admission in the poisoning unit of our emergency department. Since the verbal, eye, and motor components of GCS may be influenced by the poisoned patients’ behavior in an attempted suicide, this study was designed to evaluate the values of GCS and its components in the outcomes prediction of patients with MDP [**2**,**3**].

Another simplification of the Trauma Score was developed for field triage and added information on the tarsal injury [**10**-**11**]. The CRAMS Scale (CRAMS stands for Circulation, Respiration, Abdominal/ thoracic, Motor, and Speech) was first proposed as a simplified method of field triage. It measures five components: circulation, respiration, abdominal injury and motor and speech responses. In CRAMS, a value is assigned to each variable, depending on whether it is normal (2), mildly abnormal (1), or severely abnormal (0). Scores of 9 or 10 are defined as minor trauma, e.g., patient discharged home, and scores of 8 or less are defined as major trauma, e.g. patient died in the emergency department or went to the operating room for general surgery or neurosurgery. In field tests, CRAMS accurately discriminated between major and minor trauma, with 92 percent sensitivity and 98 percent specificity. Both the retrospective and prospective studies have shown that the CRAMS method of triage is accurate in identifying the major trauma victims with a relatively high specificity and sensitivity and it is easy to use.

6. ***Disease specific*** [e.g. Ranson’s criteria for acute pancreatitis, subarachnoid hemorrhage assessment by using the World Federation of Neurosurgeons score, and liver failure assessment by using Child-Pugh or model for end stage liver disease (MELD) scoring]. 

**Trauma-Related Injury Scoring System (TRISS; 1987)**

• Overcomes the lack of comorbid conditions and physiologic variables missing from AIS/ ISS 

o Incorporates ISS, age, and 3 physiological variables

o Final modifying equation is applied depending on whether the trauma was blunt or penetrating

• Benefits 

o Has become the most accepted means of estimating trauma-related survival probabilities

o Overcomes the ISS limitations of lack of physiological and comorbid disease data

**Revised Trauma Score (RTS)**

• Measures physiologic derangement

• Combines coded values for systolic BP (SBP), respiratory rate (RR) & GCS

• Coded values are given for each GCS, SBP, RR

• Coded values can be added for score of 0-12 for the use in pre-hospital triage. 

o RTS value < 11 suggests the need for transfer to trauma center.

• Values are weighted for the in-hospital use as it follows: 

o RTS = 0.9368 (GCS coded) + 0.7326 (SBP coded) + 0.2908 (RR coded)

o Range of values 0-7.84

o RTS >5, >90% survival

o RTS < 3, < 20% survival

o Used primarily as a research tool for the outcome assessment & quality assurance

The **Poisoning Severity Score** has been elaborated, tested, and gradually revised during a project running 1991-1994. Fourteen poison centers from various countries have participated. Each center independently graded 371 cases of acute poisoning by ten different toxic agents. The data were then analyzed and compared.

The concordance in grading the severity was increased during the study period and in the last phase. There was an acceptable concordance among centers in 80% or more of the cases. Given the condition and quality of the original case records, this result was considered satisfactory and the agreement was reached on the scoring scheme presented here. 

The Poisoning Severity Score grades severity as (0) none, (1) minor, (2) moderate, (3) severe, and (4) fatal poisoning. It is intended to be an overall evaluation of the case, taking into account the most severe clinical features. The use of the Poisoning Severity Score, normally requires a follow-up of all cases, but may be used on admission or other times during the course of poisoning if this is clearly stated when data are presented [**5**-**7**].

With the emphasis on the affluence and with the increase in the longevity of the population in the developed world, there is a general interest in the way of achieving the “goodness” of life, sometimes called life satisfaction or quality of life [**8**,**9**]. There has been a rapidly increasing interest in both the generic and disease-specific measures of quality of life. The rapid growth of an industry of standardized scale development in relation to quality of life reflects the international emphasis on the provision of effective, evidence based health care, and measurement of the outcome of care in the broadest sense. The quality of life as a measure of outcome, redirects attention towards the consideration of the impact of the condition, and the treatment, on the patient’s emotional and physical functioning and lifestyle. Quality of life indicators help answer the question of whether the treatment leads to a life worth living, by providing a more patient-led baseline against which the effects of the intervention can be evaluated. Measuring the quality of life of the patient is inevitably difficult. Questions about the sensitivity, reliability, validity and generalizability of data continue to be raised because of the complex nature of diseases, treatments, expected recovery times, and, of course, the concept of quality of life itself.

## Conclusions

1. The use of the scores in the emergency medicine, toxicology and other areas of intensive medicine have become increasingly efficient.

2. Creating a prognostic score for the acute intoxications to be used by the personnel from the Emergency Departments may have positive effects in the management of the poisoned patients (e.g. the admission in a certain treatment space: cases expected to have a trend towards worsening will be directed in the resuscitation space and after a short period of time admitted in the appropriate facility; this way, the bed occupancy time in the Emergency Department will be shortened).
